# Balancing Autonomy and Safety: Developing and Pilot Testing DIGNITY Program in Rural Nursing Homes

**DOI:** 10.1093/geroni/igaf122.2066

**Published:** 2025-12-31

**Authors:** Liza Behrens, Marie Boltz

## Abstract

Person-centered care that honors individual preferences enhance the health and well-being of nursing home (NH) residents living with Alzheimer’s disease and related dementias (ADRD). However, preferences such as going outside independently are often seen as “risky” by NH staff and subsequently restricted. There is an urgent need for shared decision-making strategies to help NH staff balance perceived health and safety risks with the residents’ right to make autonomous choices, particularly in under-resourced rural NH communities. This symposium will present the preliminary results of a multi-site cluster randomized controlled pilot study to co-design with key stakeholders (Stage 1a), implement, and assess (Stage 1b) the acceptability and feasibility of an innovative risk management program: DIGNITY (Decision making in aging and dementia for autonomy). The first presentation will describe the early development of DIGNITY intervention, establishing the rationale and theoretical underpinnings of the staff-focused behavioral intervention. The second presentation will highlight the program manual and tool development within formal consultation with a community advisory board from the perspective of an attending physician. The third presentation will present results from community engagement studios with rural NH stakeholders from the perspective of a NH leader. The fourth presentation will share a case study implementing the DIGNITY process in a risky preference situation commonly encountered in all NH communities. Finally, the session will conclude with a fifth presentation reporting acceptability, feasibility, and preliminary efficacy of implementing DIGNITY within four rural NH communities.

